# Complement Factor H-Related 3 Enhanced Inflammation and Complement Activation in Human RPE Cells

**DOI:** 10.3389/fimmu.2021.769242

**Published:** 2021-11-08

**Authors:** Nicole Schäfer, Anas Rasras, Delia M. Ormenisan, Sabine Amslinger, Volker Enzmann, Herbert Jägle, Diana Pauly

**Affiliations:** ^1^ Department of Ophthalmology, University Hospital Regensburg, Regensburg, Germany; ^2^ Department of Orthopaedic Surgery, Experimental Orthopaedics, Centre for Medical Biotechnology (ZMB), University of Regensburg, Regensburg, Germany; ^3^ Chemistry Department, Al-Balqa Applied University, Al-Salt, Jordan; ^4^ Institute of Organic Chemistry, University of Regensburg, Regensburg, Germany; ^5^ Department of Ophthalmology, University Hospital of Bern and Department of Biomedical Research, University of Bern, Bern, Switzerland; ^6^ Experimental Ophthalmology, Philipps-University Marburg, Marburg, Germany

**Keywords:** AMD, complement activation, complosome, FHR-3, inflammation, oxidative stress epitopes, RETC-2, RPE cells FHR-3 alters RPE cell complosome

## Abstract

Complement Factor H-Related 3 (FHR-3) is a major regulator of the complement system, which is associated with different diseases, such as age-related macular degeneration (AMD). However, the non-canonical local, cellular functions of FHR-3 remained poorly understood. Here, we report that FHR-3 bound to oxidative stress epitopes and competed with FH for interaction. Furthermore, FHR-3 was internalized by viable RPE cells and modulated time-dependently complement component (C3, FB) and receptor (C3aR, CR3) expression of human RPE cells. Independently of any external blood-derived proteins, complement activation products were detected. Anaphylatoxin C3a was visualized in treated cells and showed a translocation from the cytoplasm to the cell membrane after FHR-3 exposure. Subsequently, FHR-3 induced a RPE cell dependent pro-inflammatory microenvironment. Inflammasome NLRP3 activation and pro-inflammatory cytokine secretion of IL-1ß, IL-18, IL-6 and TNF-α were induced after FHR-3-RPE interaction. Our previously published monoclonal anti-FHR-3 antibody, which was chimerized to reduce immunogenicity, RETC-2-ximab, ameliorated the effect of FHR-3 on ARPE-19 cells. Our studies suggest FHR-3 as an exogenous trigger molecule for the RPE cell “complosome” and as a putative target for a therapeutic approach for associated degenerative diseases.

**Graphical Abstract:**
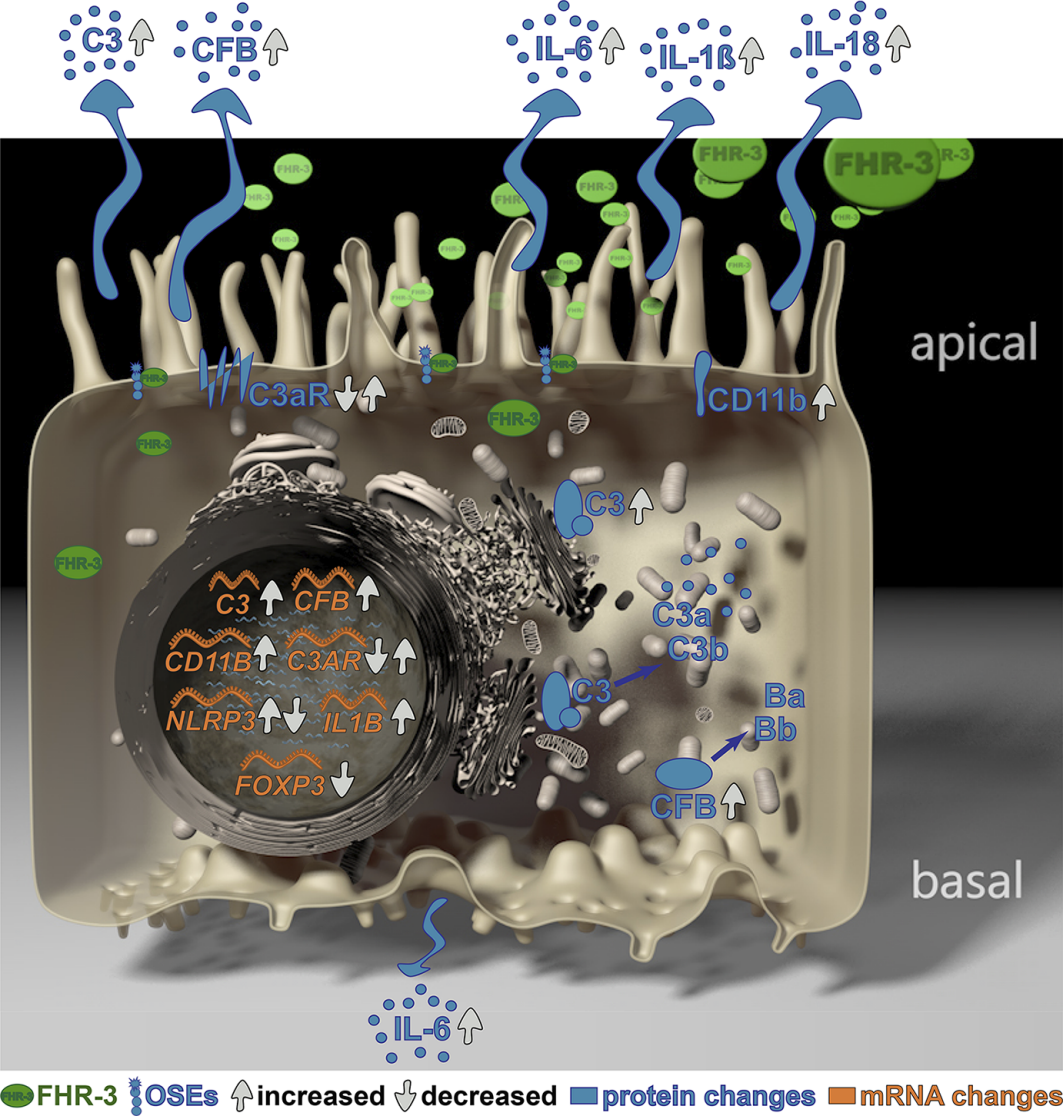


## Introduction

Studied for more than one century, but still not fully understood – the evolutionary ancient complement system is one of the first defense line of our body to protect from pathogens, cellular debris or dead cells. The complement system is tightly regulated to prevent dysregulation leading to progression of different degenerative diseases such as age-related macular degeneration (AMD) (1). One major group of soluble regulators are complement factor H (FH), its splice variant factor H-like protein 1 (FHL-1), and its related proteins 1 – 5 (FHR-1 – 5) (2–4). Single nucleotide polymorphisms (SNP) located in the *complement factor H (CFH)* gene locus have been described to be responsible for AMD pathology (5, 6). While the most relevant *CFH* SNP (Y402H) contributed to AMD progression, a joint deletion of the *complement factor-H related 1* and *3* (*ΔCFHR3/1*) genes was protective for the disease (7, 8). FHR-1 and FHR-3 are mainly produced by hepatocytes and released into the systemic circulation. In the eye, a local expression could be reported for FHR-3 by retinal macrophages/microglia cells (9) and for FH and FHL-1 by retinal pigment epithelium (RPE) cells (10).

Retinal macrophages/microglia and RPE cells are important to maintain the physiological homeostasis of the neuronal tissue. The RPE forms the outer blood-retinal barrier isolating the retina from the systemic immune system. Besides absorption of light, recycling of photoreceptor outer segements and protection against photooxidation, the RPE supports the retina by secretion of various nutrients, which is precisely regulated. However, during AMD progression age-dependent disruption and loss of RPE cells occur (11, 12). This is accompanied by loss of RPE function, e.g. failure to degrade proteins, inflammaging or increased cellular oxidative stress (13, 14).

Oxidative stress can lead to structural modifications resulting in novel oxidation-specific epitopes (OSE) detected in RPE cells (15, 16). OSE can trigger inflammation in the retina, which was inhibited by FH binding (17, 18). FHR-1 and FHR-3 also attach to OSE on necrotic cells and compete with FH for binding (19). This interference of FHR-1 and FHR-3 prevents FH-mediated proteolysis of the central complement component C3 into iC3b and results in enhanced activity of the soluble complement system (20).

Besides its blood-related function, FH also acts on complement components in the cell, the so called “complosome” (21, 22). FH is internalized by stressed and apoptotic RPE cells (18, 21), resulting in reduced endogenous C3-cleavage and increased cell survival in stressed cells (18), but contrary increased C3b-fragments are detected in apoptotic RPE cells, facilitating RPE cell opsonization (21). A similar interaction was also described for FHR-1 on necrotic cells, including RPE cells, but a cellular incorporation of FHR-1 hadn’t been reported (20, 23). Cellular FHR-1 interaction increases complement activation as well as deposition of C3 and C4 fragments on cell surfaces (23, 24). Further, inflammasome reactivity is triggered on monocytes by FHR-1 binding but not upon FHR-3 interaction (20).

In RPE cells, inflammasome activation is so far mainly related to stress and complement receptor signaling (25–27), but not to FHR-binding. RPE cells autonomously produce and activate numerous complement components independently from blood-derived complement proteins (26, 27), which can than bind to complement receptors. This local complement expression and secretion by RPE cells is modulated by external stress (27–31). Stress-related activation of the inflammasome is associated with a pro-inflammatory secretion phenotype of RPE cells (25, 30, 32, 33), which in return correlates with complement activation (30).

However, a direct effect of FHR-3 on RPE cellular physiology and its non-canonical functions modulating the “complosome” has not been described so far. Knowing that FHR-3 is expressed by retinal macrophages/microglia cells in a degenerated human retina (9), we explored the functional role of apical FHR-3 on RPE cells in depth. Here, we describe a local function of FHR-3 as an exogenous danger molecule for RPE cells, sensing those cells for “complosome” activation independent of other extracellular complement sources and inducing inflammatory immune responses. This effect was attenuated by blocking FHR-3 using a specific chimerized monoclonal antibody RETC-2-ximab.

## Materials and Methods

### Human Material and Ethical Statements

The research complies with the human research act (HRA) stating that small quantities of bodily substances removed in the course of transplantation may be anonymized for research purposes without consent (HRA chapter 5, paragraph 38, Switzerland). Human primary retinal pigment epithelial cells (hpRPE) were prepared from two anonymized donor eyes as described below (34).

Complement-depleted human sera were purchased from Complement Technology. All human serum samples were stored at −80°C.

### PCR for Genetic Analysis

DNA was isolated from human adult retinal pigment epithelium cells (ARPE-19 cells, American Type Culture Collection, #CRL-2302) lysates and sclera slices of donor eyes using ReliaPrep™ FFPE gDNA Miniprep System (Promega, Mannheim, Germany, #A2351). PCR amplification of relevant AMD-associated complement SNPs was performed using in-house generated primers (**Supplementary Table 1**) and the following cycle steps, according to the MIQE guidelines: denaturation (95°C, 1 min), annealing (60°C, 1 min), elongation (72°C, 1 min), 33 cycles. Afterwards, DNA-sequencing was performed by GeneArt (Thermo Fisher Scientific, Dreieich, Germany) using either forward or reverse in-house primers, respectively (**Supplementary Table 1**).

### Cell Culture and Treatment

ARPE-19 cells (passage 38; passage 25 for **Figures S1D–G**) were cultivated in cell culture flasks with DMEM/F12 (Sigma Aldrich, Munich, Germany), 10% foetal calf serum (FCS; PanBiotech, Aidenbach, Germany) and 1% penicillin/streptomycin until they reached confluency of approx. 80% (37°C, 5% CO_2_). Human primary RPE (hpRPE) cells were harvested from the eyecup after enzymatic digestion with dispase (1 mg/ml) and DNAse (12.2 µg/ml) for 1 h at 37°C. hpRPE cells were centrifuged at 259 × g for 5 min at 4°C and cultivated in Dulbecco’s modified Eagle’s medium/Nutrient Mixture F-12 (DMEM/F-12 GlutaMax, Thermo Fisher Scientific, #31331-028) containing 5 % FCS (Thermo Fisher Scientific, #10500-064), 1 % Penicillin-Streptomycin (Thermo Fisher Scientific, #15070-063), 1 % N1 Medium Supplement (Merck, Darmstadt, Germany, #N6530), 10 mM MEM on-essential amino acids (Thermo Fisher Scientific, #11140-035), 0.25 mg/ml taurine (Merck, #T0625), 4.5 mg/ml glucose solution (Thermo Fisher Scientific, #G524940-01), 0.013 ng/ml triiodothyronine (Merck, #T2877), 0.02 µg/ml hydrocortisone (Merck, #H0888), 20 ng/ml human basic growth factor (hbFGF, R&D Systems, Minneapolis, MN, USA, #13256029), and 1 mg/ml human epidermal growth factor (hEGF, Thermo Fisher Scientific, #PHG0311) in laminin-coated Transwell^®^ inserts under standard conditions (37°C, 5% CO_2_, 80% humidity). ARPE-19 cells were cultivated in laminin-coated Transwell^®^ inserts for 4 – 6 weeks, as published previously (30).

Before treatment, cells were cultivated in FCS-reduced medium within 3 days (5% - 2.5% - 1.25% - 0%). Afterwards, polarized ARPE-19 cells and hpRPE cells were treated apically with serum-free medium containing either: a) 50 µg/ml (1 µM) FHR-3-strep (recombinant, in-house) (9), b) FHR-1-strep (recombinant, in-house), c) FH (native, Complement Technology, Tyler, TX, USA, #A137), d) Properdin (FP, native, Complement Technology, #A139, e) 50 µg/ml (1 µM) FHR-3 and 150 µg/ml (1 µM) anti-FHR-3 RETC-2-ximab (recombinant, in-house), or f) 50 µg/ml (1 µM) FHR-3 and 150 µg/ml (1 µM) anti-BSA control-ximab (recombinant, in-house), at different time points (see figure legends).

### Immunofluorescence

**Figure 1:**
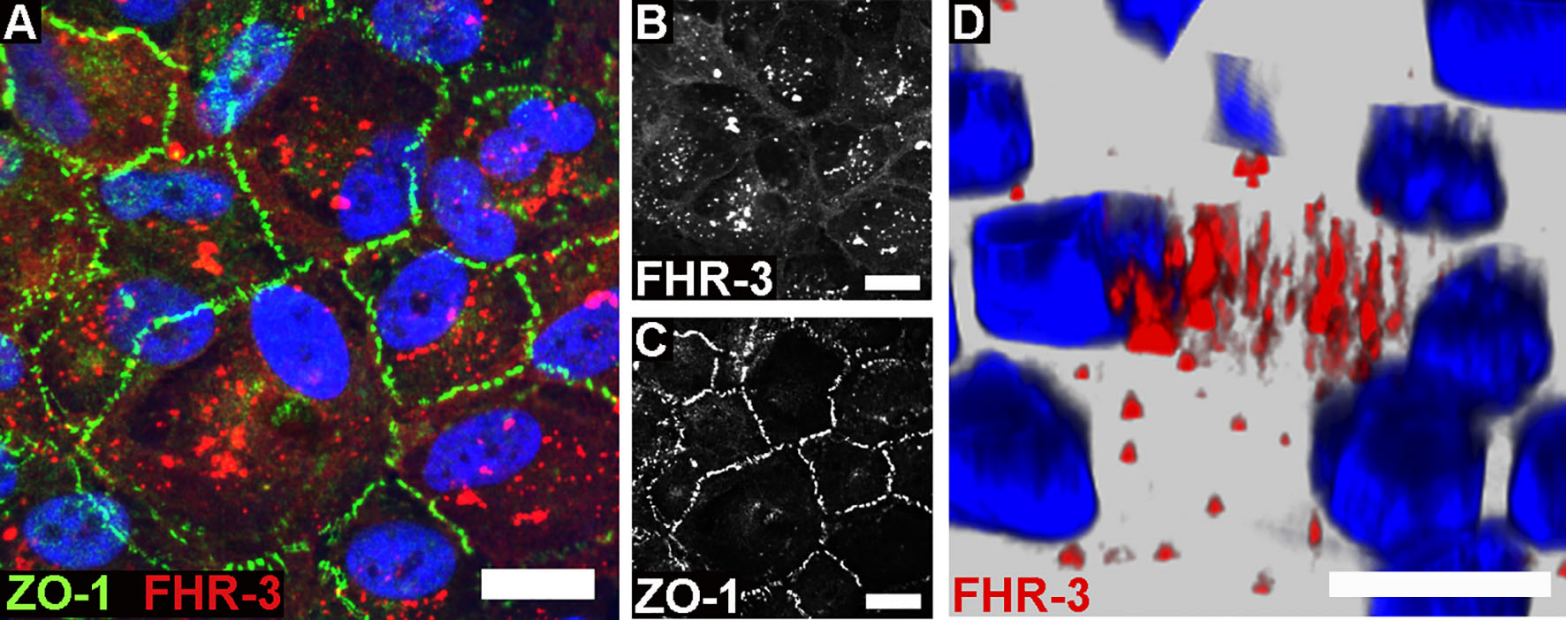
FHR-3 was internalized by ARPE-19 cells. Polarized, differentiated ARPE-19 cells were apically treated with FHR-3. **(A)** FHR-3 was specifically detected after 2 h incubation time by anti-FHR-3 antibody RETC-2 [red, **(B)**], cell-cell connections were labelled with anti-ZO-1 antibody [green, **(C)**]. Magnification 200x. **(D)** FHR-3-pHrodo (red) was specifically detected inside high-passage ARPE-19 cells. Magnification 400x. Scale bars 40 μm. Cell nuclei were stained with Hoechst.

After treatment, polarized ARPE-19 cells in Transwell^®^ inserts were permeabilized (PBS/0.2% Tween20 (PBS-T), 45 min) and paraformaldehyde-fixated (4%, 20 min). Afterwards, unspecific binding was blocked (3% bovine serum albumin (BSA)/PBS-T, 1 h). Antigens were detected using specific primary antibodies (3% BSA/PBS-T, overnight): anti-C3 (**Figures 3D, E**, **Supplementary Figure 5A**, Abcam, Cambridge, UK, #ab181147), anti-C3a desArg (**Figure 4C**, Hycult, Uden, Netherlands, #HM2074), anti-FB (**Figures 5D, E**, Merck, #341272), anti-C3aR (**Figure 6C**, Antibodies-online, Aachen, Germany, #ABIN682213), anti-cluster of differentiation molecule 11B (CD11b, CR3 subunit) (**Figure 7C**, Biorbyt, Cambridge, UK, #orb19554), anti-FHR-3 (**Figures 1A–C**, RETC-2, in-house), anti-GM130 (**Figure 3D, E**, R&D Systems, #AF8199-SP) and anti-ZO-1 (**Figures 1A, C** and **Supplementary Figure 5**, Thermo Fisher Scientific, #61-7300). Fluorescence-conjugated secondary antibodies for detection were the following (3% BSA/PBS, 45 min): anti-mouse IgG-488 (Jackson ImmunoResearch, West Grove, PA, USA, #715-545-150), anti-mouse IgG-Cy3 (Jackson ImmunoResearch, #715-165-150), anti-goat IgG-Cy3 (Jackson ImmunoResearch, #305-035-003), anti-rabbit IgG-488 (Jackson ImmunoResearch, #711-545-152) and GAPDH-HRP (Cell Signaling Technology, Beverly, MA, USA, #3683). The fluorochrome HOECHST 33342 (Thermo Fisher Scientific, #H3570) was used to stain DNA and acti-stain™ 488 phalloidin (**Figures 5D, E**, Cytoskeleton, Denver, CO, USA, #PHDG1-A) detected specifically actin filaments in ARPE-19 cells. Cells were covered with fluorescence mounting medium (Dako, Agilent Technologies, Boeblingen, Germany, #S302380-2). Images were taken with a VisiScope CSU-X1 Confocal System (Visitron Systems, Puchheim, Germany) and a high-resolution sCMOS camera, and further processed with Adobe Photoshop CC 2019.

FHR-3 labelling for internalization studies was performed using pHrodo™ Red Microscale Labeling Kit (**Figure 1D**, Thermo Fisher Scientific, #P35363) according to the manufacturer’s protocol. Labelled FHR-3 (FHR-3-pHrodo) was incubated apically on ARPE-19 cells for 60 min, cells were then fixed with paraformaldehyde (4%, 20 min). Internalized FHR-3 was detected immunohistologically by means of a Leica AF6000LX fluorescence microscope, equipped with a Leica DFC350 FX digital camera. The images were visualized by 3D modeling using Leica deconvolution software module, and further processed with Adobe Photoshop CC 2019.

### Transepithelial Resistance and Cellular Capacitance

Transepithelial resistance (TER), a measure of cell monolayer barrier function, and cellular capacitance (C_Cl_), an indicator of the expression of membrane folding such as microvilli, of polarized ARPE-19 cells were recorded online using the established cellZscope device (nanoAnalytics, Münster, Germany), as described previously (30).

### RT-qPCR

RNA was isolated using a NucleoSpin^®^ RNA/Protein kit (Macherey-Nagel, Düren, Germany). Sample quality and purity were ensured by randomly testing using QIAxcel^®^ RNA analyses (Qiagen, Hilden, Germany). Purified mRNA was transcribed into cDNA with a QuantiTect^®^Reverse Transcription Kit (Qiagen, #205313). Transcripts of complement components, receptors, and inflammation-associated markers were analyzed using a Rotor-Gene SYBR^®^Green PCR Kit either with QuantiTect Primer Assays (Qiagen, **Supplementary Table 1**) or in-house-designed primer pairs (Metabion, Planegg, Germany, **Supplementary Table 1**) in a Rotor Gene Q 2plex cycler (Qiagen), using the following conditions: hold (95°C, 5 min), cycling (95°C, 5 sec; 60°C, 10 sec; 72°C, 20 sec; 40 cycles). Data were normalized to *GAPDH* housekeeper expression, analysed by using the 2^-ΔΔCT^ method. Values were depicted on a linear scale using log_2_-transformed scores to equally visualize increases and decreases in expression levels.

### Synthesis of 2-(ω-Carboxyethyl) Pyrrole Bovine Serum Albumin

2-(w-carboxyethyl) pyrrole (CEP)-BSA was prepared using a method by Lu et al. (35), which was shortened by one step using succinic anhydride together with the Grignard reagent of 2-(2-bromoethyl)-1,3-dioxane to get directly 6-(1,3-dioxan-2-yl)-4-oxohexanoic acid in 57% yield.

The pyrrole content in CEP-BSA was determined by Ehrlich assay using the Ehrlich reagent 4-(dimethylamino)benzaldehyde and 3-(1-methyl-1*H*-pyrrol-2-yl)propanoic acid (Sigma Aldrich) as the pyrrole standard measuring the absorbance of the formed pyrrole adduct at 540 nm.

### ELISA for Oxidative Stress Epitopes Interaction

**Figure 2:**
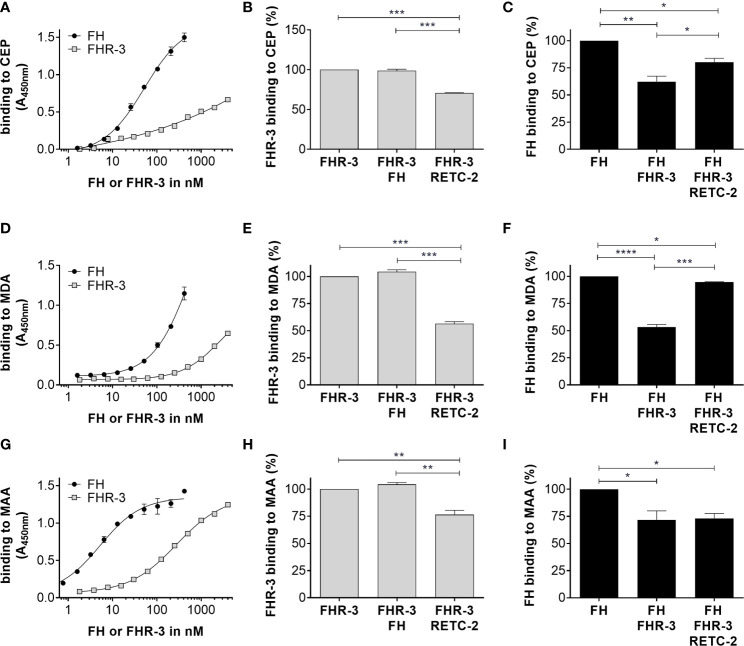
FHR-3 and FH interacted with OSEs and RETC-2 reduced this competitive binding. All three lipid peroxidation products **(A)** CEP, **(D)** MDA, and **(G)** MAA interacted concentration dependently with FHR-3 and FH. FH (black) showed a higher binding strength than FHR-3 (grey) to the OSE. Interaction of FHR-3 with **(B)** CEP, **(E)** MDA, or **(H)** MAA could not be reduced by additional incubation with FH, but RETC-2 significantly reduced the binding of FHR-3 to all three peroxidation products. Interaction of FH to **(C)** CEP, **(F)** MDA, and **(I)** MAA was significantly reduced by additional incubation with FHR-3, and anti-FHR-3 antibody RETC-2 was able to reverse this inhibitory effect completely (**F**, MDA), partially (**C**, CEP), or not at all (**I**, MAA). ****p < 0.0001, ***p < 0.001, **p < 0.01, *p < 0.05 (one-way ANOVA with Dunnett’s multiple comparisons test, n = 3).

MaxiSorp microtiter plates (Nalgene Nunc, Rochester, NY, USA) were coated either with CEP-BSA, malondialdehyde (MDA)-BSA (Hölzel Diagnostika, Köln, Germany, #20P-MD-BS102) or malondialdehyde-acetaldehyde (MAA)-BSA (10 μg/ml, PBS, overnight, 4°C). Blocking was performed with PBS-T (1 h). CEP-BSA plates (**Figures 2A–C**) were incubated with FHR-3 (200 nM), FH (6.4 nM), or with anti-FHR-3 antibody RETC-2 (666 nM). MDA-BSA plates (**Figures 2D–F**) were incubated with FHR-3 (700 nM), FH (64.5 nM), or with anti-FHR-3 antibody RETC-2 (2333 nM). MAA-BSA plates (**Figures 2G–I**) were incubated with FHR-3 (7 nM), FH (0.64 nM), or with anti-FHR-3 antibody RETC-2 (233 nM). All proteins were diluted in PBS and incubated for 1 h. FHR-3 and anti-FHR-3 antibody RETC-2 were preincubated for 1 h. For the standard curves antigen serial dilutions (FHR-3 1.8 – 4000 nM, FH 0.8 – 416 nM) were incubated (PBS, 1 h). Binding was detected either with mouse anti-FH (R&D Systems, #AF4999, PBS, 1 h) and anti-mouse IgG-Fcγ-POD (Jackson ImmunoResearch, #115-035-164, PBS, 30 min) or StrepMAB-HRP (IBA Lifesciences, Goettingen, Germany, #2-1509-001, PBS, 30 min) to detect the FHR-3-strep. The signal was developed with 3,3’,5,5’-Tetramethylbenzidine (TMB, Seramun Diagnostica, Heidesee, Germany, #S-004-4), and absorption was determined at 450 nm.

### Expression of recombinant FHR-3 and FHR-1

FHR-3 with a Strep-tag II was expressed in HEK293 cells (American Type Culture Collection) as previously described (10).

FHR-1 was transiently expressed in HEK293 cells. For this, the *CFHR1* gene was cloned into expression vector pEXPR-IBA103 containing a c-terminal Strep-tag II (IBA) by using specific in-house-designed primer pairs (Metabion, **Supplementary Table 1**). The generated construct pEXPR-CFHR1 was transiently inserted into HEK293 cells (Thermo Fisher Scientific) with TransIT-LT1 Transfection Reagent (Mirus Bio, Madison, WI, USA, #MIR2305), according to the manufacturer’s protocol. FHR-1 with Strep-tag II was purified from HEK293 supernatants using Strep-Tactin Sepharose columns (IBA). After gradient elution of FHR-1, the recombinant protein was concentrated by vacuum centrifugation. Protein purity was detected with Coomassie staining and Western blot using a specific anti-FHR-1 antibody (R&D Systems, #MAB4247). Strep-tagged FHR-1 was used as a control protein to exclude an off-side effect of the Strep-tag on FHR-3-treated RPE cell inflammation and complement expression.

### Protein Secretion Assays

FHR-3 and properdin levels in cell culture supernatants were determined using sandwich ELISA, as described previously (9, 36). Complement secretion levels of ARPE-19 cell supernatants were determined using MILLIPLEX MAP Human Complement Panel 1 and 2 (Merck, #HCMP1MAG-19K, HCMP2MAG-19K). Cytokine concentrations were determined according to the protocol of a custom ProcartaPlex^®^ multiplex immunoassay kit (Thermo Fisher Scientific, #PPX-05). The readout of the multiplex assays was performed using a Magpix instrument (Luminex, Austin, TX, USA). Vascular endothelial growth factor (VEGF)-α concentrations were determined using a human VEGF Quantikine ELISA Kit (R&D systems).

### Western Blot

**Figure 3:**
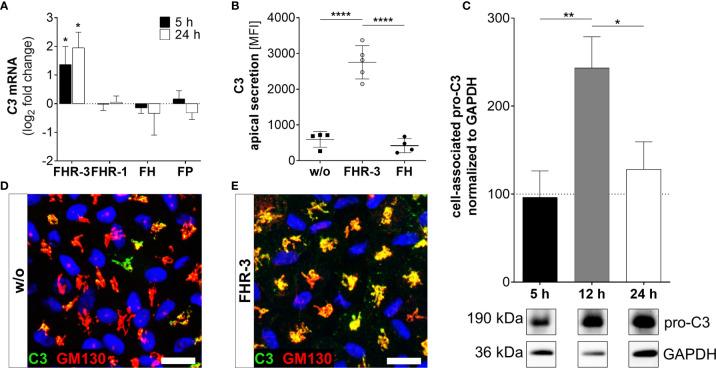
FHR-3 increased C3 expression and secretion in ARPE-19 cells. **(A)** *C3* mRNA expression was increased 5 h and 24 h after apical treatment of ARPE-19 cells with FHR-3, but not after incubation with FHR-1, FH or FP, respectively. This effect could be confirmed at the protein level showing an FHR-3-dependent increase **(B)** in C3 protein secretion after 24 h, **(C)** elevated pro-C3 protein (190 kDa) in ARPE-19 cell lysates in Western blots after 12 h, and **(D, E)** increased intracellular C3 protein levels by immunofluorescence using anti-C3 (green) and anti-GM130 (red, cis-Golgi marker) antibodies after 12 h treatment. C3 was co-localized with the cis-Golgi (yellow). Scale bars 40 µm. w/o untreated control [**(A, C)** dotted line]. Mean with standard deviation is shown. Full Western blot in **Supplementary Figure 1K**. ****p < 0.0001, **p < 0.01, *p < 0.05. **(A)** Wilcoxon matched-pairs signed rank test (n = 3); **(B, C)** Ordinary one-way ANOVA, Turkey’s multiple comparisons test (n = 3).

**Figure 4:**
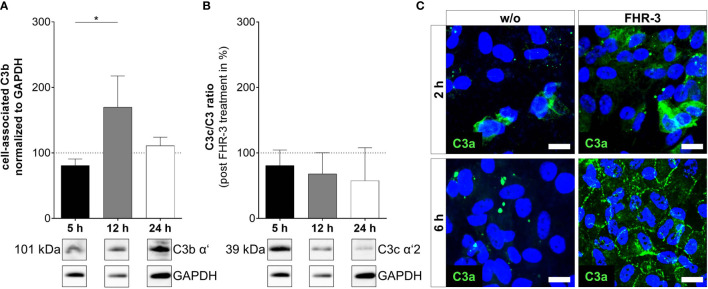
C3 cleavage products were changed in ARPE-19 cells after FHR-3 incubation. **(A)** Increased C3b levels (101 kDa) 12 h after FHR-3 treatment and **(B)** a time-dependent reduction of C3c fragments (39 kDa) in ratio to pro-C3 were detected in Western blots of ARPE-19 cell lysates. **Supplementary Figure 1K** shows full Western blots. **(C)** Anaphylatoxin C3a was detected by immunofluorescence using a specific anti-C3a antibody (green). C3a increased time-dependently from 2 h to 6 h after FHR-3 treatment and was translocated from the cytoplasm (upper right panel) to the cell membrane (lower right panel). Scale bars 40 µm. w/o untreated control (dotted line). Mean with standard deviation is shown. *p < 0.05. **(A, B)** Ordinary one-way ANOVA, Turkey’s multiple comparisons test (n = 3).

**Figure 5:**
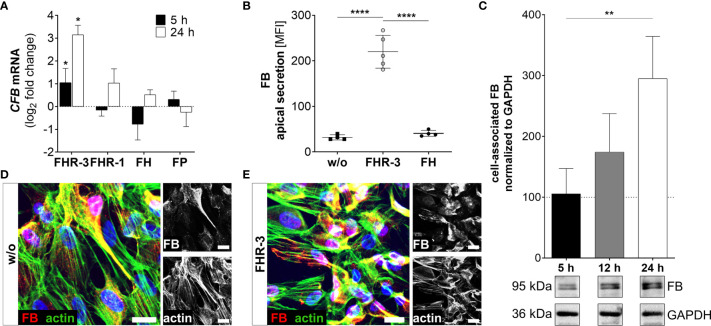
FHR-3 increased FB expression and secretion in ARPE-19 cells. **(A)** *CFB* mRNA increased after apical FHR-3 treatment of ARPE-19 cells, but not following incubation with FHR-1, FH or FP. This effect could be confirmed at the protein level: **(B)** Apical FB secretion was increased 24 h after FHR-3 incubation. **(C)** Western blots of ARPE-19 cell lysates detected a time-dependent increase in FB levels (95 kDa) 24 h after FHR-3 treatment. **Supplementary Figure 1L** shows full Western blots. **(D, E)** Increased FB protein levels were detected by immunofluorescence using specific anti-FB (red) and anti-actin (green) antibodies 12 h after FHR-3 treatment. FB was co-localized partly with actin stress fibers (yellow). Scale bars 40 µm. **(A–C)** w/o untreated control (dotted line). **(A–C)** Mean with standard deviation is shown. ****p < 0.0001, **p < 0.01, *p < 0.05. **(A)** Wilcoxon matched-pairs signed rank test (n = 3); **(B, C)** Ordinary one-way ANOVA, Turkey’s multiple comparisons test (n = 3).

**Figure 6:**
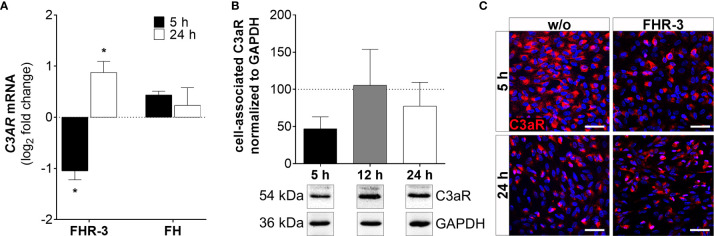
C3aR expression was time-dependently regulated in FHR-3 stressed ARPE-19 cells. **(A)** *C3AR* mRNA expression was decreased after 5 h and increased after 24 h FHR-3 incubation. This effect could be confirmed on protein level: **(B)** Western blots of ARPE-19 cell lysates showed a tendency for decreased C3aR levels (54 kDa) 5 h after FHR-3 treatment. **Supplementary Figure 1M** shows full Western blots. **(C)** Decreased C3aR protein levels were detected by immunofluorescence using a specific anti-C3aR antibody (red) after 5 h FHR-3 incubation (upper panels), whereas no differences between FHR-3 stressed and unstressed controls were observed after 24 h (lower panels). Scale bars 40 µm. w/o untreated control (dotted line). Mean with standard deviation is shown. *p < 0.05. **(A)** Wilcoxon matched-pairs signed rank test (n = 3); **(B)** Ordinary one-way ANOVA, Turkey’s multiple comparisons test (n = 3).

**Figure 7:**
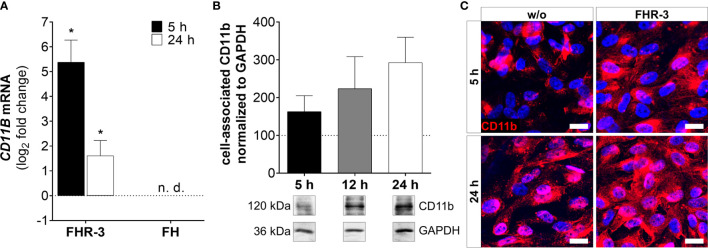
CD11b expression was highly elevated in FHR-3 treated ARPE-19 cells. **(A)** *CD11B* mRNA expression was highly elevated after 5 h and 24 h FHR-3 incubation. No expression of *CD11B* mRNA could be detected when cells were treated with FH. The effect of FHR-3 could be confirmed at the protein level: **(B)** Western blots of ARPE-19 cell lysates showed a time-dependent increase in CD11b levels (120 kDa) from 5 h – 24 h after FHR-3 incubation. **Supplementary Figure 1N** shows full Western blots. **(C)** Elevated CD11b protein levels were detected by immunofluorescence using a specific anti-CD11b antibody (red) after 5 h and 24 h of FHR-3 incubation (right panels). Scale bars 40 µm. w/o untreated control (dotted line). n.d. not detected. Mean with standard deviation is shown. *p < 0.05. **(A)** Wilcoxon matched-pairs signed rank test (n = 3); **(B)** Ordinary one-way ANOVA, Turkey’s multiple comparisons test (n = 3).

ARPE-19 cell lysates were dissolved in RIPA buffer (Sigma-Aldrich, #R0278) with protease and phosphatase inhibitors (1:100, Sigma-Aldrich, #P8340). Samples were diluted in reducing ROTI^®^Load 1 (Carl Roth, Karlsruhe, Germany, #K929.1) and denatured (95°C, 10 min). Following sample separation in a 12% SDS-PAGE, proteins were transferred onto an activated polyvinylidene difluoride membrane using a wet-blotting system. Membranes were blocked with 5% BSA/TBS-T (1 h) and incubated with specific primary antibodies (5% BSA/TBS-T, overnight): anti-C3b-α (**Figures 3C**, **4A, B**, Progen, Heidelberg, Germany, #61019), anti-FB (**Figure 5C**, Merck, #341272), anti-C3aR (**Figure 6B**, Antibodies-online, #ABIN682213), anti-CD11b (**Figure 7B**, Biorbyt, #orb19554), anti-GAPDH. Detection was performed using peroxidase-conjugated anti-species antibodies (TBS, 1 h): anti-mouse IgG-Fcγ-POD (Jackson ImmunoResearch, #115-035-164), anti-goat IgG-Fcγ-POD (Jackson ImmunoResearch, #305-035-003), anti-rabbit IgG-Fcγ-POD (Jackson ImmunoResearch, #111-035-003), anti-GAPDH-HRP (Cell Signaling Technology, #3683). Visualization was performed by WesternSure PREMIUM Chemiluminescent Substrate (LI-COR, Bad Homburg, Germany) in a Fluor Chem FC2 Imaging System (Alpha Innotech, San Leandro, CA, USA). Afterwards, Western blots were quantified against a GAPDH housekeeping protein and calculated using Image Studio Lite (LI-COR).

### Chimerization of Anti-FHR-3 Antibody RETC-2

Variable regions (heavy and light chain) of murine RETC-2 (anti-FHR-3) and murine control antibody (anti-BSA) were determined as described previously (9) and cloned into plasmids containing either human IgG4 heavy chain (pFUSE-CHIg-hG4e1, *In vivo*gen, Toulouse, France, #pfuse-hchg4e1) or human IgG kappa light chain (pFUSE2-CLIg-hk, *In vivo* gen, #pfuse2-hclk), according to the manufacturer’s protocol. The generated constructs for the heavy and light chains were transiently co-transfected into HEK293 cells (Thermo Fisher Scientific, using IgG-free FCS in Medium) using a ratio of 3 : 2 (light chain construct : heavy chain construct) with TransIT-LT1 Transfection Reagent (Mirus), according to the manufacturer’s protocol. Cell supernatants were tested for antibody amount and for specific FHR-3 binding using ELISA and Western blots. Chimerized antibodies were purified using Protein-A columns HiTrap^®^ MabSelect^™^ SuRe^™^ (Merck, #GE11-0034-94) and named as follows: RETC-2-ximab, control-ximab.

### Statistics

Statistics were performed using GraphPad Prism 8 (GraphPad Software Inc., San Diego, CA, USA). All data are expressed as mean ± standard deviation (SD) unless stated otherwise. Detailed information about specific n-values, implemented statistical analyses and coding of significance levels are documented in each figure and figure legend, respectively.

## Results

We previously reported that FHR-3 is co-localized with activated macrophages/microglia cells in an aged and inflamed retina, but not detectable in a healthy retina (9). Due to our additional results, showing that RPE cells in response to stress increased the expression of complement components and pro-inflammatory factors (30), we proposed that FHR-3 could be a stress factor for the RPE in the aged retina promoting retinal degeneration (9). Consequently, we investigated here the cell-specific complement and inflammation-associated response of RPE cells exposed to FHR-3.

To exclude a genotype-primed reactivity ARPE-19 cells and human primary RPE cells (hpRPE) were used in this study. We determined the most common AMD-associated SNPs within genes of the complement pathway in these cells (37). Homogenous AMD-risk SNPs could not be detected in the examined RPE cells, instead heterozygous SNPs were present in the *CFH* and *C3* gene of ARPE-19 and *CFH*, *C2/CFB*, *CFI* and *ARMS* gene of hpRPE cells (**Supplementary Figure 1A**).

We verified the epithelial phenotype of the used ARPE-19 cells, passaged for 38 times and cultivated under *in vivo*-like conditions, by staining tight junction protein *zonula occludens 1* (ZO-1). A polarized monolayer could be detected for untreated and FHR-3 treated cells, showing that FHR-3 had no effect on stable cell-cell contacts. We also excluded that ARPE-19 cell passaging had any influence on FHR-3 dependent tight junction formation by comparing ZO-1 stainings in passage number 38 (P38) with 25 (P25) (**Supplementary Figures 1D–G**). Transepithelial resistance and cellular capacitance of the polarized ARPE-19 cells were measured between 0.5 – 72 h of FHR-3-treatment. FHR-3 had no impact on cell barrier function and on cell membrane folding (**Supplementary Figure 1B**). These characterizations resulted in a specific RPE phenotype, with a slight shift to mesenchymal characteristics established by mRNA expression analysis of epithelial-mesenchymal transition (EMT) markers vimentin (*VIM*), α‐smooth muscle actin (*ACTA2*) and collagen type 1 (*COL1A1*) (**Supplementary Figures 1H–J**). FHR-3 treatment increased *VIM* expression in ARPE-19 cells P38 and P25 compared to untreated controls (**Supplementary Figure 1H**), whereas *ACTA2* and *COL1A1* were only raised in ARPE-19 cells P25 (**Supplementary Figures 1I, J**) indicating an early EMT caused by FHR-3 and already existing mesenchymal characteristics in high-passage ARPE-19 cells (P38).

Due to the number of passages and the slight EMT, which is typical for aged human RPE cells (38), the investigated ARPE-19 cells at P38 were used in this study.

### FHR-3 Was Internalized by ARPE-19 Cells

Previous reports described an interaction of proteins of the FH-protein family with damaged cells: FH was internalized by apoptotic ARPE-19 cells (21), FHR-1 and FHR-5 bound to necrotic ARPE-19 cells (23), and FHR-1 and FHR-3 interacted with a necrotic-type endothelial cell line (19, 20). In this study, we showed that FHR-3 was bound to and internalized by viable ARPE-19 cells (**Figure 1** and **Supplementary Figure 1C**), knowing that *CFHR3* mRNA is not expressed in these cells (**Supplementary Figure 3**). Interaction of FHR-3 with ARPE-19 cells was confirmed by immunofluorescence resulting in FHR-3-positive ARPE-19 cells following FHR-3 incubation (**Figures 1A, B**). Further, in the supernatant of the ARPE-19 cells supplemented with FHR-3 only 30% of the added FHR-3 remained in the apical and 5% in the basal supernatant compared to the added complement control protein properdin (FP), which was stable to 92% in the apical and 3% in the basal supernatant after incubation with ARPE-19 cells (**Supplementary Figure 1C**). To investigate whether the complement regulator was internalized, we labelled FHR-3 with pH-sensitive dye pHrodo (FHR-3-pHrodo). This dye is non-fluorescent outside the cell, but fluoresces in acidic, cellular compartments like endosomes, phagosomes or lysosomes. Added FHR-3-pHrodo was detected inside high-passage ARPE-19 cells indicating a phagocytosis or receptor-mediated endocytosis (**Figure 1D**). This shift in fluorescence activity could not be detected when cells were incubated with FH-pHrodo concluding that FH was not internalized in viable ARPE-19 cells.

Just recently, it was shown that FHR-3 binds to malondialdehyde (MDA)-epitopes (19). These OSE are present on the surface of stressed ARPE-19 cells (16). We determined as well specific binding of FHR-3 and FH either to OSE 2-(ω-carboxyethyl)pyrrole (CEP), MDA or malondialdehyde-acetaldehyde (MAA) (**Figures 2A, D, G**). We detected a competitive binding of FHR-3 and FH to CEP, MDA and MAA. Interaction of FHR-3 and oxidative stress epitopes was not changed by additional incubation of FH (**Figures 2B, E, H**), whereas a reduced FH interaction with CEP (38%), MDA (47%) and MAA (28%) could be observed when FHR-3 was added as a competitor (**Figures 2C, F, I**). Our previously published anti-FHR-3 antibody RETC-2 (9) decreased binding of FHR-3 to CEP (29%), MDA (44%) and MAA (24%) (**Figures 2B, E, H**), and prevented the competitive effect of FHR-3 with FH for the interaction with CEP (47%) and MDA (87%). This impact could not be detected for MAA (**Figures 2C, F, I**).

Here, we showed FHR-3 internalization by viable, high-passage ARPE-19 cells (**Figure 1**), and determined peroxidation products CEP, MDA and MAA as potential ligands. FHR-3 bound to OSE and prevented their interaction with FH, which was reversed by anti-FHR-3 antibody RETC-2 (9) (**Figure 2**).

### FHR-3 Increased Endogenous Complement Activation

Complement components are locally expressed by different retinal cell types and by ARPE-19 cells (27, 30, 31, 39). Here, we showed that the complement regulator FHR-3 enhanced complement expression and secretion of RPE cells (**Figures 3** **–** **5** and **Supplementary Figure 2A**). We demonstrated an increase of *C3* mRNA expression in polarized high-passage ARPE-19 cells and hpRPE cells after FHR-3 treatment, whereas no expression change was shown for FHR-1, FH or FP incubation (**Figure 3A**, **Supplementary Figure 2A**). On protein level, an increased C3 secretion after 24 h (**Figure 3B**) and enhanced cell-associated precursor form of C3 protein (pro-C3, 190 kDa) detection in cell lysates after 12 h and 24 h FHR-3 incubation compared to untreated cells were examined (**Figure 3C** and **Supplementary Figure 1K**). We visualized accumulated intracellular C3, co-localized with Golgi complex, which seems to be mainly pro-C3, increased in 12 h FHR-3-treated ARPE-19 cells (**Figures 3D, E**).

In line with our previous published data, where we reported C3 activation fragments in ARPE-19 cells under oxidative stress (30), we assume an indirect involvement of FHR-3 in C3 cleavage in ARPE-19 cells due to increase of C3 protein concentration (**Figure 4**). However, we did not detect a direct contact of FHR-3 and C3 in ARPE-19 cells (**Supplementary Figure 5**), nor an increase in *cathepsin L* (*CTSL*) expression, which cleaves intracellular C3 (**Supplementary Figure 3**) (40). We detected raised levels of cell-associated C3b (101 kDa) after 12 h of FHR-3 treatment (**Figure 4A**). However, C3c as a marker for inactivated C3 was time-dependently reduced (5 h – 24 h) when ARPE-19 cells were incubated with FHR-3 (**Figure 4B**). Anaphylatoxin C3a was increased in FHR-3-treated cells and showed a translocation from the cytoplasm to the cell membrane after FHR-3 exposure (**Figure 4C**).

Increased detection of FB was also a consequence of FHR-3 addition to ARPE-19 cells and hpRPE cells (**Figure 5** and **Supplementary Figure 2B**). *CFB* transcripts were highly elevated after 5 h and 24 h of FHR-3 incubation and no expression changes were shown for FHR-1, FH or FP treatment (**Figure 5A** and **Supplementary Figure 2B**). Enhanced FB protein secretion after 24 h (**Figure 5B**) and a time-dependent increase in cell-associated FB protein expression in cell lysates from 5 h to 24 h of FHR-3 treatment were determined (**Figure 5C**). FB cleavage products Bb and Ba were also detected in ARPE-19 cells and raised in FHR-3-treated cells (**Supplementary Figure 1L**). Using immunofluorescence, we showed accumulated intracellular FB, partly co-localized with actin stress fibers in FHR-3 treated ARPE-19 cells (**Figures 5D, E**).

Our data indicated a cell-associated complement-activating effect of FHR-3 by enhancing C3 and FB expression as well as secretion and by anaphylatoxin C3a increase in immortal ARPE-19 cells (**Figures 3**–**5** and **Supplementary Figure 4**) as well as cultivated, post-mitotic, hpRPE cells (**Supplementary Figures 2A, B**).

### FHR-3 Altered Complement Receptor Expression of ARPE-19 Cells

ARPE-19 cells express a variety of complement receptors (30, 41). It has recently been reported, that oxidatively stressed ARPE-19 cells increased expression of complement receptors CR3 and C5aR1 (30). Here, we showed that FHR-3 modified complement receptor C3aR (**Figure 6** and **Supplementary Figure 2C**) and CD11b (**Figure 7** and **Supplementary Figure 2D**) expression on high-passage ARPE-19 cells and hpRPE cells, independently from any systemic complement.

*C3AR* mRNA expression was time-dependently changed with decreased transcripts after 5 h (**Figure 6** and **Supplementary Figure 2C**) and elevated expression after 24 h of FHR-3 exposure (**Figure 6A**). This was partly in accordance with protein data showing decreased cell-associated C3aR levels in Western blots (**Figure 6B**) and immunohistochemically (**Figure 6C**) 5 h after FHR-3 incubation. An increase in C3aR protein expression 24 h after treatment, as shown at the mRNA level, could not be verified. This might be explained by storage of *C3AR* RNA into RNA granules, either stress granules or processing bodies (42).

ARPE-19 cells express the α-chain CD11b of the integrin complement receptor CR3 (30). We detected very low mRNA expression in untreated-, and no expression in FH-treated ARPE-19 cells. When cells were incubated with FHR-3, *CD11B* expression increased significantly in ARPE-19 and hpRPE cells, respectively (**Figure 7A** and **Supplementary Figure 2D**). Western blot analyses revealed a time-dependent upregulation of CD11b from 5 h – 24 h after FHR-3 incubation (**Figure 7B**). Immunostainings of FHR-3 treated and untreated cells confirmed CD11b accumulation after 5 h and 24 h (**Figure 7C**). We did not observe a change in *C5aR1* mRNA transcript levels following FHR-3 treatment, and *C5aR2* mRNA transcripts were not detected in ARPE-19 cells (**Supplementary Figure 3**).

These results revealed an FHR-3 triggered alteration of complement receptor C3aR and CD11b expression in immortal, high-passage ARPE-19 cells (**Figures 6**, **7** and **Supplementary Figure 4**) as well as cultivated, post-mitotic, hpRPE cells (**Supplementary Figures 2C, D**).

### FHR-3 Induced Pro-Inflammatory Markers in ARPE-19 Cells

**Figure 8:**
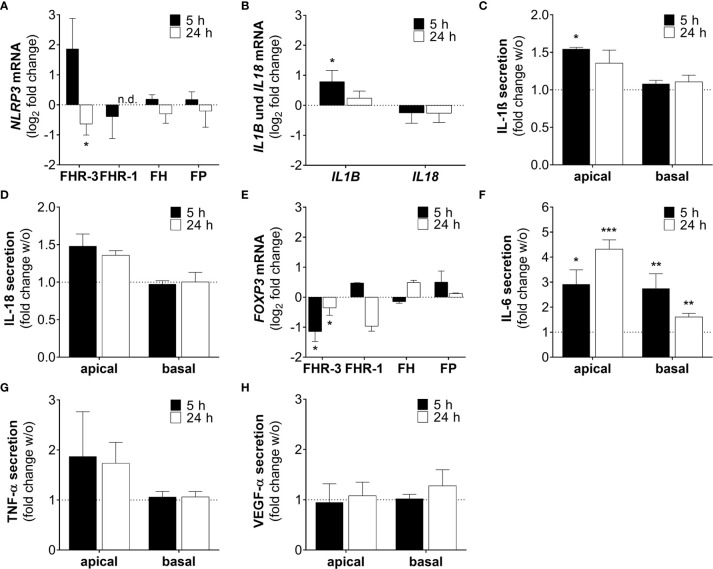
FHR-3 induced an ARPE-19 cell dependent pro-inflammatory microenvironment. **(A)** *NLRP3* mRNA expression in ARPE-19 cells was either increased or decreased after 5 h or 24 h FHR-3 treatment, respectively. This was in contrast to no expression changes following FHR-1, FH or FP incubation. **(B)** Transcripts of *IL1B* were significantly elevated, whereas *IL18* mRNA expression was slightly decreased after FHR-3 treatment. **(C, D)** Apical secretion levels of **(C)** IL-1ß and **(D)** IL-18 were increased in ARPE-19 cells after FHR-3 stress. **(E)** *FOXP3* mRNA expression was reduced 5 h and 24 h after FHR-3 incubation. **(F)** Secretion of IL-6 protein was significantly raised after FHR-3 incubation, both on the apical and basal cell site. **(G)** ARPE-19 cell secretion of TNF-α protein was on trend increased after FHR-3 incubation on the apical site compared to w/o. **(H)** VEGF-α was secreted by ARPE-19 cells, but showed no differences between FHR-3 treated and untreated cells. w/o untreated control (dotted line). n.d. not detected. Mean with standard deviation is shown. ***p < 0.001, **p < 0.01, *p < 0.05. **(A, B, E)** Wilcoxon matched-pairs signed rank test (n = 3); **(C, D, F–H)** Unpaired t-test (n = 5).

NLRP3 inflammasome activation is considered as an additional hallmark for the development of AMD (43). It has already been shown that anaphylatoxins C3a and C5a, and oxidative stress lead to the priming of NLRP3 and secretion of pro-inflammatory cytokines in ARPE-19 cells (25, 30, 44). Here, we describe that FHR-3, a potential risk factor of AMD progression, induced inflammasome-associated pro-inflammation in ARPE-19 cells (**Figures 8A–D**). *NLRP3* mRNA expression was elevated 5 h and slightly decreased 24 h after FHR-3 incubation, whereas treatment with FHR-1, FH or FP did not show any expression changes (**Figure 8A**). Accordingly, transcripts of *IL1B* were also upregulated 5 h and 24 h after FHR-3 incubation (**Figure 8B**). In line with this, determination of cytokine secretion levels of FHR-3-treated ARPE-19 cells revealed an upregulation of apical secretion of IL-1ß (**Figure 8C**) and IL-18 (**Figure 8D**) compared to untreated cells (**Figures 8C, D**, dotted line).

Inflammatory cellular micro-environments are shaped by further key players, e.g. forkhead box P3 (FOXP3), an inflammation-associated transcription factor, which triggers secretion of anti-inflammatory cytokines in regulatory T-cells. Expression of *FOXP3* was detected recently in ARPE-19 cells (30, 45, 46). We determined a time-dependent significant decrease in *FOXP3* mRNA expression, after FHR-3 incubation (**Figure 8E**), indicating a pro-inflammatory effect of FHR-3.

Additionally, the concentration of the pro-inflammatory cytokine IL-6 was elevated in both, apical and basal supernatants of FHR-3-incubated ARPE-19 cells (**Figure 8F**). A tendency to higher secretion levels of tumor necrosis factor (TNF)-α could be also detected in apical supernatants of ARPE-19 cells incubated with FHR-3 (**Figure 8G**). However, a regulation of pro-angiogenic marker vascular endothelial growth factor (VEGF)-α, which is important for proper RPE function, could not be determined after FHR-3 incubation (**Figure 8H**).

In sum, these results proposed a pro-inflammatory role of FHR-3 on aged RPE cells, independent from blood-derived complement components, which may have a so far unknown impact on AMD progression (**Supplementary Figure 4**).

### RETC-2-ximab Diminished The Inflammatory Effect of FHR-3

**Figure 9:**
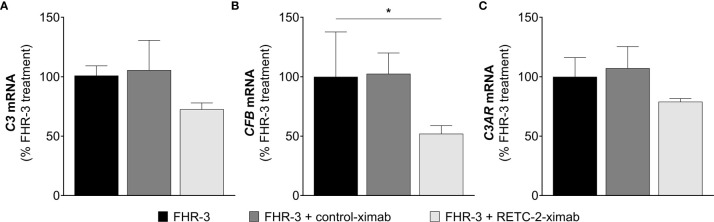
RETC-2-ximab slightly ameliorated the effect of FHR-3 on ARPE-19 cells. ARPE-19 cells were treated with either FHR-3 (black), FHR-3 and control-ximab (anti-BSA, dark grey) or FHR-3 and RETC-2-ximab (anti-FHR-3, light grey). **(A)** *C3* mRNA expression was reduced by 27%, **(B)** *CFB* mRNA expression was reduced by 48%, and **(C)** *C3AR* mRNA expression was reduced by 21% when incubated with FHR-3 and RETC-2-ximab compared to FHR-3 alone. Shown is the relative mRNA expression to FHR-3 treated cells in %. Mean with standard deviation is shown. *p < 0.05. Unpaired t-test (n = 2).

In our previous studies we generated a highly specific monoclonal mouse antibody (mAb) against human FHR-3, RETC-2. We showed that RETC-2 inhibits binding of FHR-3 to C3b and regained interaction of FH to C3b (9). Similar results could be observed regarding FHR-3 and FH binding to OSEs (CEP, MDA, MAA), as described afore (**Figure 2**). To further investigate a therapeutic potential of RETC-2, chimerization was performed to replace complement-activating mouse regions of the mAb. The chimerized anti-FHR-3 mAb, called RETC-2-ximab, was tested in *in vitro* studies with polarized high-passage ARPE-19 cells, which were treated apically for 24 h with FHR-3, FHR-3 and RETC-2-ximab or with FHR-3 and an antibody isotype control (control-ximab, specific for BSA) (**Figure 9**). Gene expression of *C3* decreased by 27% (**Figure 9A**), *CFB* was reduced significantly by 48% (**Figure 9B**), respectively, and *C3AR* mRNA expression was decreased by 21% (**Figure 9C**) after combined treatment with FHR-3/RETC-2-ximab compared to FHR-3 alone or with the FHR-3/control-ximab in high-passage ARPE-19 cells.

These promising results could offer a potential recovery of local complement homeostasis and a reduced progression of retinal degeneration using RETC-2-ximab in the future.

## Discussion

It has been known for 15 years, that the deletion of the genes for *CFHR3* and *CFHR1* (Δ*CFHR3/1*) is associated with a protective effect for the development of AMD (7, 47). However, the local biological function of FHR-3 and FHR-1 proteins is still poorly understood. We discovered recently, that FHR-3 – not expressed in RPE cells – is produced by macrophages/microglia within an aged, inflamed retina (9). In this study, we focussed on the intraocular role of FHR-3 on RPE cells and did not consider whether FHR-3 could interact basally with the RPE to examine a systemic effect, as Cipriani et al. recently described for FHR-4 (48).

Here, we demonstrated a complement activating and pro-inflammatory effect on human RPE cells mediated by FHR-3, which could be ameliorated by a chimeric anti-FHR-3 antibody.

### Endocytosis of FHR-3 by Viable ARPE-19 Cells

Our data revealed an incorporation of FHR-3 by high-passage viable RPE cells. So far, internalization of other FH-family proteins has been solely described for apoptotic cells. Martin et al. examined a specific FH uptake by apoptotic rendered Jurkat T- and ARPE-19 cells, whereas neither binding nor uptake could be confirmed by living cells (21). FHR-1 and FHR-5 have also been reported to bind to necrotic RPE and endothelial cells, but an uptake was not described (20, 23).

FHR-3 uptake could be linked to OSE and potentially unknown co-receptors on aged RPE cells as potential ligands for FHR-3-dependent membrane invagination. Joseph et al. showed that oxidatively stressed RPE cells express lipid peroxidation product MDA on their cell surface (16). In accordance to Alic et al. we showed a specific binding of FHR-3 to MDA-epitopes and a competitive interaction with FH to these OSE (19). We also observed the same interaction of FHR-3 with CEP, a peroxidation product serving as biomarker for AMD (49). It is known that FH binds to OSE and thereby inhibits pro-inflammation in the aging eye (17, 18), indicating that FHR-3-FH-OSE interaction may impact the risk for AMD progression. Just recently, Irmscher et al. detected a binding of FHR-1 to MDA-epitopes (20) and Rudnick et al. showed an interaction of FHR-5 with lipid peroxidation products (50), both indicated an enhanced complement activity and inflammation in endothelial cells and monocytes. To what extend lipid peroxidation may be sufficient for cell membrane invagination and consequent uptake of FHR-3, rather than FH in viable RPE cells – even this pathway of lipid-mediated endocytosis is poorly understood (51) – needs to be further investigated.

### FHR-3, a Danger Associated Molecule Within the Eye?

Beyond the classical function of the complement system as part of the humoral immune system, which has been known for more than 100 years, more and more non-canonical functions and the “complosome” have been assigned in the last decade, including the influence on cell metabolism, cell development and regeneration (22, 52, 53). In the past years, rising data indicate that FHR proteins have complement activating properties (20, 21, 23, 24, 48, 50). While FHR-1, FHR-3, FHR-4 and FHR-5 compete systemically with FH for C3b binding, less is known about their non-canonical functions as cellular modulators (23, 48, 54, 55). Here, we treated serum-free, *in vivo*-like cultivated ARPE-19 and hpRPE cells apically with FHR-3, FHR-1, FH or properdin, respectively, to investigate the cell-associated complement and inflammatory response independent of any external complement sources.

We showed that FHR-3 is an inflammatory stimulator and may serve as a damage-associated sensing molecule for RPE cells (56). Just recently, we reported complement expression in hpRPE cells (27, 31), ARPE-19 cells (30) and murine RPE (39). Here, we described that FHR-3 enhanced complement activation in cultured RPE cells. C3 cleavage was indicated by increased C3b protein detection and accumulation of C3a in ARPE-19 cells specifically following FHR-3 treatment, but not with FHR-1, FH or properdin incubation. Based on our results we suggest FHR-3 as a modulator of cellular C3/FB protein concentration mediated by an unknown ligand-receptor signal transduction. Contrary, Martin et al. showed that apoptotic ARPE-19 cells – not cultivated under *in vivo*-like conditions – internalized FH leading to increased cleavage and deposition of endogenous C3 (21). Previous studies investigating oxidative stress in ARPE-19 cells also reported increased C3 accumulation and secretion of C3 and C3a (30, 57).

We also showed a time-dependent redistribution of C3a to the cell membrane in FHR-3 treated ARPE-19 cells. C3a, especially *via* C3aR signaling, is known to disrupt RPE function and promote AMD progression (58). In a previous study, C3a overexpression leaded to formation of sub-RPE deposits which are associated with early-stage AMD (59). Local C3a effects could be also reported in other cells: Liszewski et al. described that intracellular lysosomal C3a led to the activation of the C3aR, which was essential for the survival of T cells (40). In endothelial cells, C3a overexpression disrupted barrier integrity *via* C3aR-signaling (60).

Our data revealed FHR-3 as a cellular stressor for RPE cells, which could balance the physiological and inflammatory state of the outer retinal-blood barrier.

Asgari et al. described that C3a-mediated C3aR signaling in monocytes caused initiation of the NLRP3 inflammasome and secretion of the pro-inflammatory cytokine IL-1ß (61). Further, the C3 fragment receptor CR3, has been also associated with NLRP3 inflammasome activation in human primary RPE cells (62). Besides recently shown for ARPE-19 (30) and hpRPE cells (27), CR3 expression has been mainly associated with macrophages (63) so far.

Here, we showed that FHR-3 regulated time-dependently C3aR and CR3 expression in RPE cells. Long-term FHR-3 incubation increased complement receptor *C3AR* and *CD11b* transcript levels in ARPE-19 cells.

FHR-3 does not only possess complement-activating properties, but also triggers pro-inflammatory potentials in RPE cells. Supplementation of ARPE-19 cells with FHR-3 increased the mRNA expression of *NLRP3* and *IL1B* and, subsequently, enhanced the secretion of pro-inflammatory cytokines IL-1ß and IL-18. Irmscher et al. described an FHR-1 triggered induction of NLRP3 inflammasome in monocytes (20). Inflammasome priming in RPE cells was shown to be influenced by addition of extracellular anaphylatoxins and oxidative stress so far (25). We added FHR-3 to this list, potentially mediating NLRP3-priming by cell-autonomous C3a. However, we recently demonstrated that exogenous added properdin, the main positive regulator of the complement system, depicted an opposed effect and showed anti-inflammatory properties (27).

In line with these results, we also observed that FHR-3 increased secretion of the pro-inflammatory cytokine IL-6 by ARPE-19 cells. IL-6 plays an important role during intraocular inflammation. Previous studies demonstrated an increased IL-6 release by oxidatively stressed (64) and C3a-stimulated RPE cells (65).

FOXP3, an inflammation-associated transcription factor, promotes the secretion of anti-inflammatory cytokines in regulatory T cells (45). Busch et al. demonstrated that FOXP3 is expressed in ARPE-19 cells and activated by extracellular addition of anaphylatoxins C3a and C5a (46). Recent studies determined increased *FOXP3* mRNA expression in oxidatively stressed ARPE-19 cells (30). In the present study, *FOXP3* mRNA was decreased in FHR-3 stimulated ARPE-19 cells, which may be consistent with the pro-inflammatory effect of FHR-3 on RPE cells. However, the exact role of FOXP3 in RPE cells is not known so far.

Related to these pro-inflammatory events triggered by FHR-3, we chimerized our anti-FHR-3 antibody RETC-2 and hypothesized that RETC-2-ximab might serve as a new therapeutic approach for treatment of degenerated eye diseases such as AMD. We demonstrated that RETC-2-ximab slightly mitigated the effect of FHR-3 on high-passage ARPE-19 cells. These promising data needs to be further investigated to elucidate the potential of RETC-2-ximab restoring local complement homeostasis. Just recently, it has been shown that a local complement suppression can be an effective tool for treating geographic atrophy, a late-stage AMD. In a phase 2 trial, intravitreal injection of the C3 inhibitor Pegcetacoplan significantly reduced the geographic atrophy rate over 12 months (66).

Here, we demonstrated FHR-3 – but not FHR-1, FH or properdin – as a complement promoting and pro-inflammatory protein on human RPE cells. Our *in vitro* studies, using ARPE-19 cells and hpRPE cells, unveiled a putative local, intraocular function of FHR-3. In addition, we chimerized our highly specific monoclonal antibody against FHR-3 (9) – RETC-2-ximab – and showed promising inhibitory potential.

## Data Availability Statement

The original contributions presented in the study are included in the article/**Supplementary Material**. Further inquiries can be directed to the corresponding author.

## Ethics Statement

The research complies with the human research act (HRA) stating that small quantities of bodily substances removed in the course of transplantation may be anonymized for research purposes without consent (HRA chapter 5, paragraph 38, Switzerland). Written informed consent for participation was not required for this study in accordance with the national legislation and the institutional requirements.

## Author Contributions

Conceptualization: NS and DP. Data curation: NS and DP. Formal analysis: NS. Funding acquisition: NS and DP. Investigation: NS, AR, DMO, SA, VE, and DP. Methodology: NS, AR, SA, VE, and DP. Project administration: NS and DP. Supervision: HJ and DP. Visualization: NS and DP. Writing – original draft: NS. Writing – review and editing: NS, SA, VE and DP. All authors have read and agreed to the published version of the manuscript.

## Funding

This project was supported by the Bright Focus Foundation (Grant Reference ID: M2015186 to DP), Dr. Werner Jackstädt-Stiftung (DP), European Union’s Horizon 2020 research and innovation programme (SciFiMed, grant agreement No 899163) (DP) and by the Maloch Stiftung (NS).

## Conflict of Interest

The authors declare that the research was conducted in the absence of any commercial or financial relationships that could be construed as a potential conflict of interest.

## Publisher’s Note

All claims expressed in this article are solely those of the authors and do not necessarily represent those of their affiliated organizations, or those of the publisher, the editors and the reviewers. Any product that may be evaluated in this article, or claim that may be made by its manufacturer, is not guaranteed or endorsed by the publisher.

## Abbreviations

ACTA2, α‐smooth muscle actin; AMD, age-related macular degeneration; ARPE-19, human adult retinal pigment epithelium cells; BSA, bovine serum albumin; C_Cl,_ cellular capacitance; CD11b, cluster of differentiation molecule 11B (CR3 subunit); CEP, ω−carboxyethyl)pyrrole; COL1A1, collagen type I, alpha 1; CR3, complement receptor 3; EMT, epithelial-mesenchymal transition; FH, factor H; FHL-1, factor-H like protein 1; FHR-1 – 5, factor H-related proteins 1 – 5; FOXP3, forkhead box P3; FP, properdin; hpRPE, primary human RPE cells; MAA, malondialdehyde-acetaldehyde; MDA, malondialdehyde; NLRP3, NLR family pyrin domain containing 3; OSE, oxidation-specific epitopes; RETC-2, REgensburg Therapy Complement 2 (anti-FHR-3 antibody); RETC-2-ximab, chimerized REgensburg Therapy Complement 2 (anti-FHR-3 antibody); RPE, retinal pigment epithelium; SNP, single nucleotide polymorphisms; TER, Transepithelial Resistance; TNF, tumor necrosis factor; VIM, vimentin; ZO-1, zonula occludens 1.
