# Supplementary material for: Complement Factor H-Related 3 Enhanced Inflammation and Complement Activation in Human RPE Cells
Source: Front Immunol. 2021 Nov 8;12:769242. doi: 10.3389/fimmu.2021.769242 (PMC8606654; doi:10.3389/fimmu.2021.769242)
Supplement: Supplementary file 1 [file DataSheet_1.pdf]

Supplementary Material

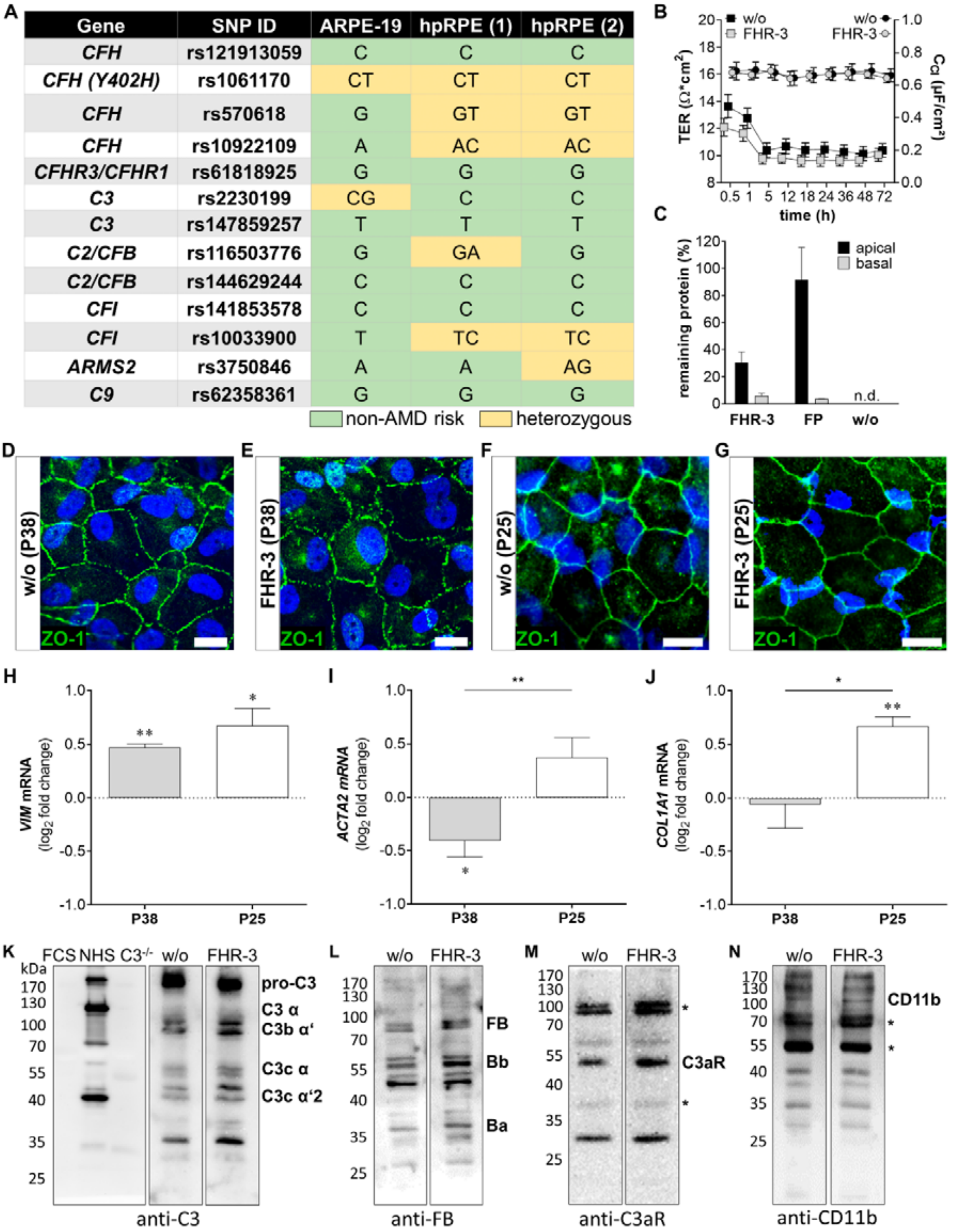

Gene	SNP ID	ARPE-19	hpRPE (1)	hpRPE (2)
CFH	rs121913059	C	C	C
CFH (Y402H)	rs1061170	CT	CT	CT
CFH	rs570618	G	GT	GT
CFH	rs10922109	A	AC	AC
CFHR3/CFHR1	rs61818925	G	G	G
C3	rs2230199	CG	C	C
C3	rs147859257	T	T	T
C2/CFB	rs116503776	G	GA	G
C2/CFB	rs144629244	C	C	C
CFI	rs141853578	C	C	C
CFI	rs10033900	T	TC	TC
ARMS2	rs3750846	A	A	AG
C9	rs62358361	G	G	G

Supplementary Figure 1: Characterization of investigated ARPE-19 and hpRPE cells.

(A) Genotyping of all used cells (ARPE-19 cells, human primary RPE cells: hpRPE (1), hpRPE (2)) was performed for the most common AMD-associated complement SNPs. All typings resulted either in non- (green) or heterozygous-AMD risk SNPs (yellow). **(B, square)** Transepithelial resistance (TER) and **(B, circle)** cellular capacitance (C_{Cl}) were measured for w/o and FHR-3 treated cells for 0.5 h – 72 h. FHR-3 had no effect on **(B, square)** TER and **(B, circle)** C_{Cl}. **(C)** ARPE-19 cells were incubated for 24 h with either FHR-3 or Properdin (FP) as control protein. Only 30% of the incubated FHR-3 could be detected in the apical supernatant, whereas 92% of apically incubated properdin was still detectable. **(D – G)** Polarized ARPE-19 cells, either **(D, E)** high-passage (P38) or **(F, G)** low-passage (P25), showed an intact monolayer when tight junction protein zonula occludens-1 (ZO-1) was immunohistochemically detected (green), in both **(D, F)** w/o and **(E, G)** FHR-3 treated cells. **(H – J)** FHR-3 increased EMT marker mRNA expression **(H)** *VIM* in high-passage ARPE-19 cells (P38), and **(H)** *VIM,* **(I)** *ACTA2,* **(J)** *COL1A1* in low-passage ARPE-19 cells (P25) 24 h after incubation compared to w/o. **(K – N)** Western Blots of ARPE-19 cell lysates detected **(K)** pro-C3 (190 kDa), C3b (101 kDa), and C3c fragments (61 kDa, 39 kDa). No detection was observed in FCS used for cell cultivation and in $C3^{-/-}$ Serum, **(L)** FB (95 kDa), and cleavage products Bb (60 kDa) and Ba (35 kDa), **(M)** C3aR (54 kDa) and C3aR glycosylations (stars, 40 - 100 kDa), **(N)** subunit CD11b (120 kDa) of complement receptor 3 (CR3) and cleavage products (stars). All proteins could be observed in w/o and FHR-3 treated cells, under serum-free conditions (exemplarily shown 5 h post-treatment). FCS fetal calf serum. NHS normal human serum. $C3^{-/-}$ C3-depleted human serum. Scale bars 40µm. w/o untreated control (dotted line). Mean with standard deviation is shown. $^{**}p < 0.01$, $^{*}p < 0.05$. (H – J) unpaired t-test (n = 3); (C) interpolated against the respective standard curve.

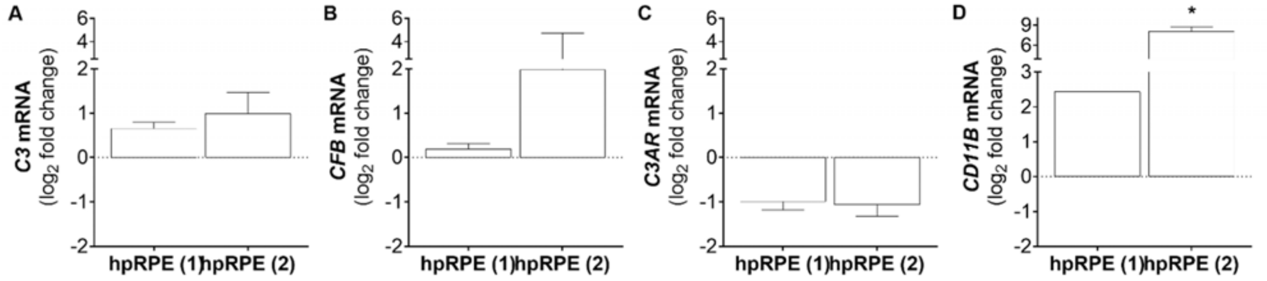

Supplementary Figure 2: FHR-3 increases mRNA expression of *C3*, *CFB*, *C3AR* and *CD11B* in hRPE cells.
(A) *C3*, **(B)** *CFB* and **(D)** *CD11B* mRNA expression was increased in two different human primary RPE cells (hpRPE (1), hpRPE (2)) after FHR-3 incubation (5 h) compared to the untreated controls (dotted line). **(C)** Expression of *C3AR*, in contrast, was decreased. *$p < 0.05$. Wilcoxon matched-pairs signed rank test ($n = 3$).

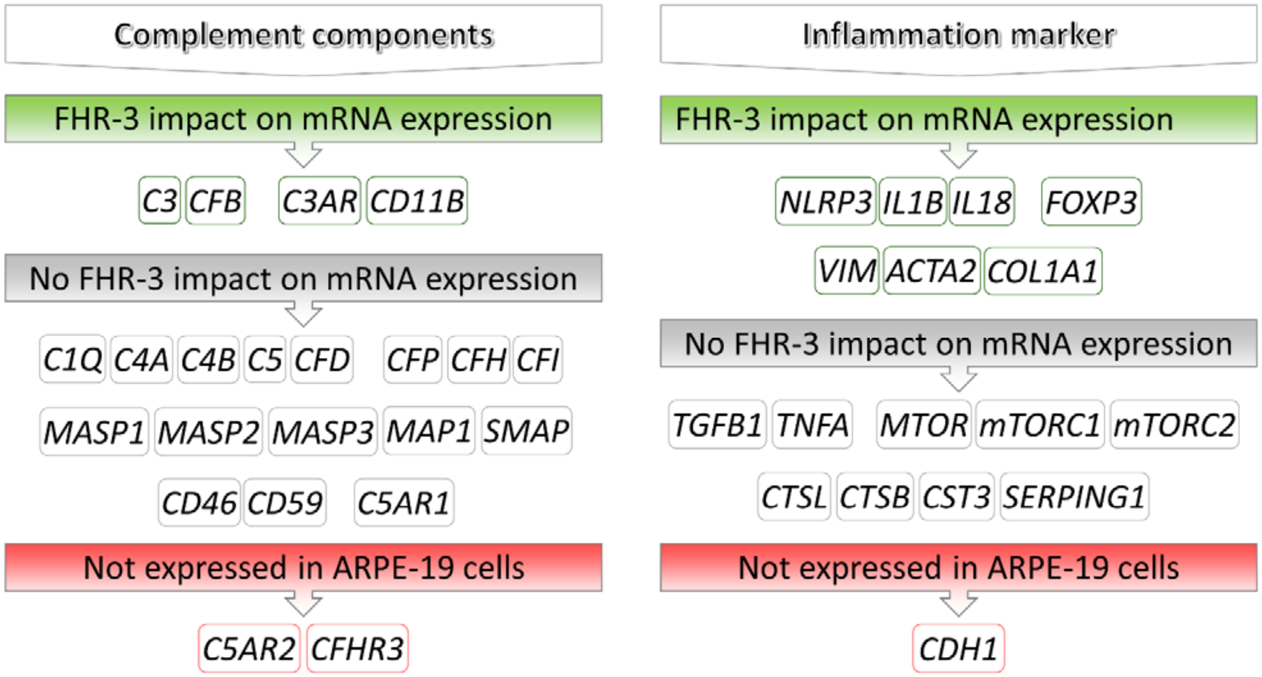

Supplementary Figure 3: Overview of investigated mRNA expression in ARPE-19 cells and impact of FHR-3 on their transcript levels.
Shown in green are all transcripts whose expression has been altered due to FHR-3 treatment. In grey are all genes whose mRNA expression has not been influenced by FHR-3 and in red are all genes that are not expressed in ARPE-19 cells at the mRNA level in this study.

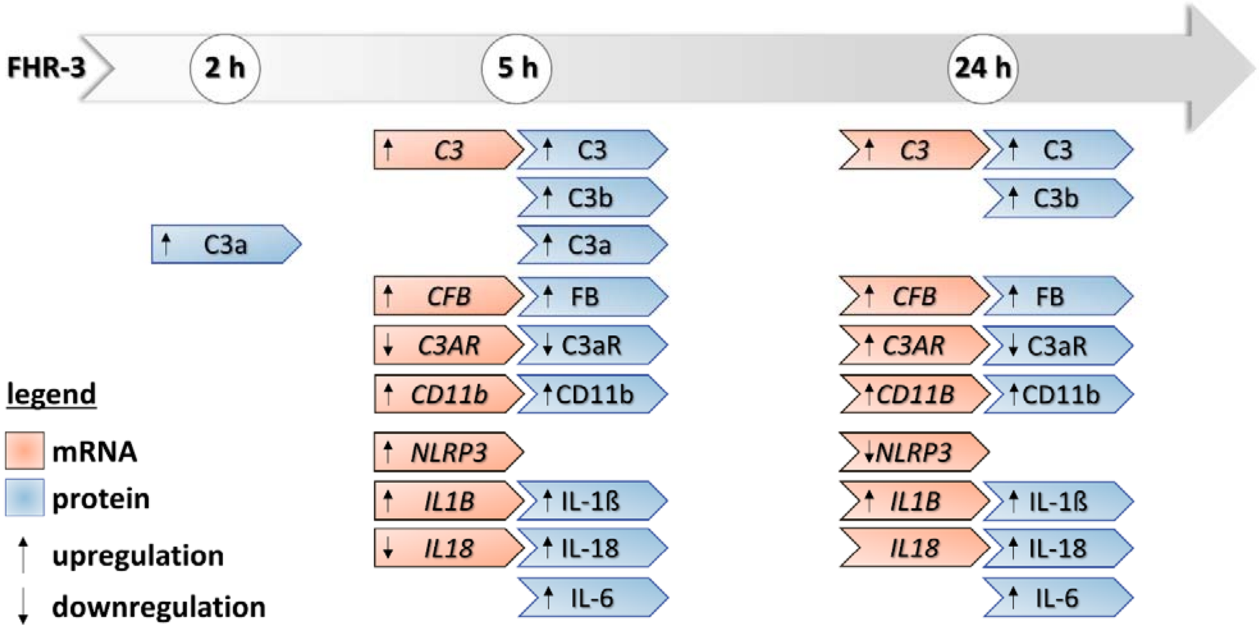

Supplementary Figure 4: Timeline of FHR-3-treated ARPE-19 cells with RNA and protein regulation of complement components and inflammation markers.

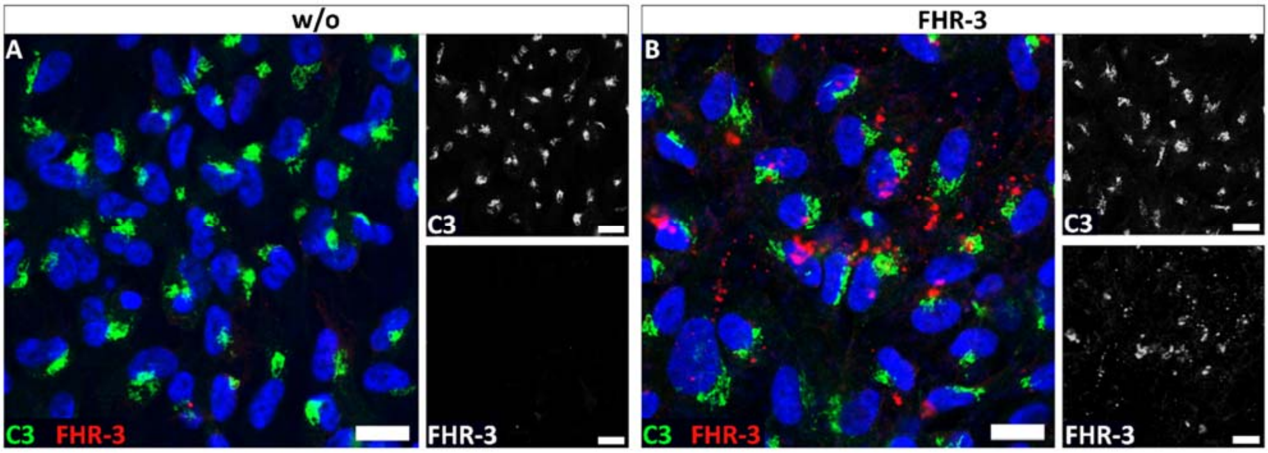

Supplementary Figure 5: FHR-3 did not co-localize with C3 in ARPE-19 cells.
Intracellular C3 (green) was detected by immunofluorescence in **(A)** untreated and **(B)** FHR-3 treated (24 h) ARPE-19 cells. FHR-3 was solely detected in **(B)** FHR-3 treated ARPE-19 cells by using anti-FHR-3 antibody RETC-2. Co-localization of C3 and FHR-3 could not be shown in ARPE-19 cells by immunofluorescence. Scale bars 40 µm. w/o untreated control.

Supplementary Table 1: PCR and RT-qPCR primers

transcript	name or sequence	Catalogue number	Usage/Company
C1QB	Hs_C1QB_1_SG	QT00003493	RT-qPCR/ Qiagen
C3	Hs_C3_1_SG	QT00089698	
C3AR	Hs_C3AR1_1_SG	QT00090398	
C4A	Hs_C4A_1_SG	QT00237160	
C5	Hs_C5AR2_1_SG	QT00243971	
C5AR1	Hs_C5R1_1_SG	QT00997766	
C5AR2	Hs_C5AR2_1_SG	QT00243971	
CD11B	Hs_ITGAM_1_SG	QT00031500	
CD46	Hs_MCP_1_SG	QT00073689	
CD59	Hs_CD59_1_SG	QT00035952	
CFB	Hs_BF_1_SG	QT00012138	
CFD	Hs_CFD_1_SG	QT00212191	
CFH	Hs_CFH_1_SG	QT00001624	
CFHR3	Hs_CFHR3_1_SG	QT00001631	
CFI	Hs_CFI_1_SG	QT00213794	
CFP	Hs_CFP_1_SG	QT00010514	
CST3	Hs_CST3_1_SG	QT00046438	
CTSB	Hs_CTSB_1_SG	QT00088641	
CTSL	Hs_CTSL_1_SG	QT01664978	
FOXP3	Hs_FOXP3_1_SG	QT00048286	
GAPDH	Hs_GAPDH_1_SG	QT00079247	
MBL2	Hs_MBL2_1_SG	QT01004227	
NLRP3	Hs_NLRP3_1_SG	QT00029771	
SERPING1	Hs_SERPING1_1_SG	QT00053963	
ACTA2	fw: GCCTTGGTGTGTGACAATGG<br>rv: AAAACAGCCCTGGGAGCAT		RT-qPCR/ in-house, Metabion
CDH1	fw: ACAGGCTGGCTGAAAGTGAC<br>rv: CACGGCATGAGAATAGAGGAT		
COL1A1	fw: GGTGGTTTCTTGGTCGGT<br>rv: ATGACGTGATCTGTGACGAG		
IL18	fw: ACTGTAGAGATAATGCACCCCG<br>rv: AGTTACAGCCATACCTCTAGGC		
IL1B	fw: CTCGCCAGTGAAATGATGGCT<br>rv: GTCGGAGATTCGTAGCTGGAT		
MAP1	fw: TGAAGGATGGGACGTGGAGT<br>rv: TGCTCTGACTTGAGTTCGCT		
MASP1	fw: TGCAAAGGTTCCCAGAGACC<br>rv: GCATAAGCCTTCTGGCAGGT		
MASP2	fw: GCGGTTTCTACTGCTCCTGCC<br>rv: CGTGGGTATTCAGGGCTGCTGAGCTC		
MASP3	fw: TGCCCAAGGAGTCTGGATGA<br>rv: CCCCCAATGATCCTCTTGACC		
MTOR	fw: TGCCAACTATCTTCGGAACC<br>rv: GCTCGCTTCACCTCAAATTC		
RAPTOR	fw: ACTGATGGAGTCCGAAATGC		

	rv: TCATCCGATCCTTCATCCTC	
RICTOR	fw: GGAAGCCTGTTGATGGTGAT<br>rv: GGCAGCCTGTTTTATGGTGT	
SMAP	fw: TACGTCCTGCACCGTAACAAG<br>rv: CAGCCATCTCAGCCCAACC	
TGFB1	fw: CATAGCTGACTTCAAGATGTGGT<br>rv: CCTAGTGAGACTTTGAACCGT	
TNFA	fw: GCTGCACTTTGGAGTGATCG<br>rv: GGGTTTGCTACAACATGGGC	
VIM	fw: TGTCCAAATCGATGTGGATGTTTC<br>rv: TTGTACCATTCTTCTGCCTCCTG	
ARMS_375<br>**(SNP ID: rs3750846)**	fw:<br>GCATTTCTAATATCAAATCAAAACATTC<br>AT<br>rv: CCTGGGGCTCTGTTTGAATTG	PCR for genetic analysis/<br>in-house, Metabion
C2_116<br>**(SNP ID: rs116503776)**	fw: AGCACCATCTACACTTCGCC<br>rv: CATGGCCCTTACCTCGACAT	
C2_144<br>**(SNP ID: rs144629244)**	fw: CAGGGGGAGATCAGAATCGTC<br>rv: GTGGGGGCAGGGAAATTCTTT	
C3_147<br>**(SNP ID: rs147859257)**	fw: TTGCCTCTCCTAAGCCTGTG<br>rv: GGCTAGGGTCTCACGAGG	
C3_223<br>**(SNP ID: rs2230199)**	fw: CTGGATGAAGAGGTACCCGC<br>rv: CTTGGAACAGACCCCTGACA	
C9_623<br>**(SNP ID: rs62358361)**	fw: TCTGTGACAATGGCAGACAACT<br>rv: TTTATTCCCCACTCTCTGTTATCTT	
CFH_106<br>**(SNP ID: rs1061170)**	fw:<br>CCTTTGTTAGTAACTTTAGTTCGTCTT<br>rv: GGAGTAGGAGACCAGCCATTC	
CFH_109<br>**(SNP ID: rs10922109)**	fw: AGTCTACCTTGCTAACGGTTC<br>rv: CCTTCTAGATTGTAGTACCTGTTGCT	
CFH_121<br>**(SNP ID: rs121913059)**	fw: CAGATCCGTGTGTAATATCCCGA<br>rv: TAAGAAGAGAGCCACCGGTCTCA	
CFH_570<br>**(SNP ID: rs570618)**	fw: AGTGGTGAGGAAAAATGTAGAATGT<br>rv: CATCTCCATCTTTCTTAAATTCTGTGA	
CFH_618<br>**(SNP ID: rs61818925)**	fw: GTGCATTCAGGGGCATATTC<br>rv: CCTGAAGACACAAATCTCACTGG	
CFHR1	fw: GGATCCATGTGGCTCCTGGTCAGTG<br>rv:<br>CTCGAGTCTTTTTGCACAAGTTGGATAC<br>TC	
CFI_100<br>**(SNP ID: rs10033900)**	fw: CCTGCAGTGTGGTCTGTGAG<br>rv: CCAGTGCTACAAGGTGGGAA	
CFI_141<br>**(SNP ID: rs141853578)**	fw: ATCCACTGATACAAGCGCTCA<br>rv: TTAAAATCGTCATGATGTTCAAAGC	
